# hMPV Lineage Nomenclature and Heparin Binding

**DOI:** 10.3390/v5102546

**Published:** 2013-10-16

**Authors:** Penelope Adamson, Sutthiwan Thammawat, Gamaliel Muchondo, Tania Sadlon, John Williams, David Gordon

**Affiliations:** 1 Department of Microbiology and Infectious Diseases, Flinders University, Flinders Medical Centre, Bedford Park, SA 5042, Australia; E-Mails: ant3714@hotmail.com (S.T.); gamaliel.muchondo@health.sa.gov.au (G.M.); tania.sadlon@health.sa.gov.au (T.S.); d.gordon@flinders.edu.au (D.G.); 2 Department of Microbiology and Infectious Diseases, SA Pathology, Flinders Medical Centre, Bedford Park, SA 5042, Australia; 3 Department of Pediatrics, Pathology, Microbiology and Immunology, Vanderbilt University Medical Center, Nashville, TN 37232-2581, USA; E-Mail: john.williams@vanderbilt.edu

**Figure 1:**
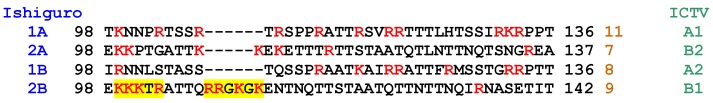
Comparison of the predicted amino acid sequence for representatives of each strain of human metapneumovirus (hMPV) G protein (residues 98–136/137/142 of the extracellular domain). Strains ordered by Ishiguro’s classification are shown in blue and strains ordered by the proposed International Committee on Taxonomy of Viruses (ICTV) nomenclature are shown in green. The number of positively charged residues (shown in red in the sequence) is indicated in brown at the end of each sequence. The yellow highlights in the 2B/B1 sequence indicate the previously identified heparin binding domains [6].

Human metapneumovirus (hMPV), first described in 2001 [1], is responsible for causing serious respiratory illness in young children, the elderly and immunocompromised patients. Four distinct lineages of hMPV have been identified with the original nomenclature for these subgroups (A1, A2, B1 and B2), reported by van den Hoogen *et al.* [2], utilised by many. An alternate terminology (1A, 1B, 2A and 2B) was also published by Ishiguro *et al.* in 2004 [3] which has been adopted by others. However, this has caused some confusion in the interpretation of publication results as the terminology is similar yet describes different subtypes. As a result, a number of investigators have made a submission to the International Committee on Taxonomy of Viruses (ICTV, ICTV taxonomic proposal 2012.012V) for the official adoption of the original terminology as an approved nomenclature for hMPV [4]. We welcome this officially approved nomenclature which should provide clarification of these subtypes in future. Therefore to assist with the interpretation of our recently published research in the 2012 special issue of Viruses: Pneumoviruses and Metapneumoviruses entitled “Diversity in Glycosaminoglycan Binding Amongst hMPV G Protein Lineages” [5] we have updated the Figure 3 in this letter (see Figure 1), showing the proposed ICTV terminology compared to the Ishiguro classification (used in our publication). Note that in the original publication the alphanumeric order for the Ishiguro classification was transposed (e.g., 1A was referred to as A1).
